# Identification and validation of an alternative splicing-based prognostic signature for head and neck squamous cell carcinoma

**DOI:** 10.7150/jca.44746

**Published:** 2020-05-18

**Authors:** Xinyuan Zhao, Shanshan Si, Xiaona Li, Wenjuan Sun, Li Cui

**Affiliations:** 1 Stomatological Hospital, Southern Medical University, Guangzhou 510280, China.; 2 Department of Stomatology, the Third Affiliated Hospital of Sun Yat-sen University, Guangzhou 510630, China.; 3 UCLA School of Dentistry, Los Angeles, CA 90095, USA.

**Keywords:** prognostic signature, head and neck squamous cell carcinoma, survival analysis, alternative splicing events, splicing factor

## Abstract

Increasing evidence has demonstrated that changes in alternative splicing (AS) events are closely associated with the initiation and progression of cancer. However, the concrete role of AS in tumorigenesis of head and neck squamous cell carcinoma (HNSCC) is poorly known. In this study, we aimed to investigate the AS profile in HNSCC, and build up a robust AS-based prognostic signature for HNSCC. Our results revealed a total of 4068 overall survival (OS) associated AS events in the TCGA HNSCC cohort. The whole TCGA HNSCC cohort was randomly divided into discovery cohort and validation cohort. A prognostic signature including five AS events was developed with the discovery cohort based on the most significant OS-associated AS events. Then it was further successfully validated in the validation cohort. The AS-based risk signature was an independent prognostic indicator in both discovery cohort and validation cohort. This prognostic signature-based nomogram model showed excellent performance for predicting the OS of HNSCC. Splicing network analysis have identified the most correlated splicing factor-AS network in HNSCC. Collectively, we have constructed a robust AS-based prognostic signature which might contribute to improve the clinical outcome of HNSCC.

## Introduction

Head and neck cancer (HNC) is the sixth most common cancer around the world, and squamous cell carcinoma (HNSCC) accounts for more than 90% of all HNC cases 1, 2. It represents a leading reason for cancer-attributable morbidity and mortality, with a global incidence of 550 000 cases and 380 000 deaths annually 3. Currently the selection of therapeutic methodologies mainly depends upon the tumor, node, metastasis (TNM) staging system, which is widely used for the predicting the prognosis of HNSCC. However, it is not reliable and accurate enough as many patients at the same TNM stage might have distinct clinical outcome. Therefore, these limitations have prompted a search for novel biomarkers which can precisely predict the overall survival (OS) of HNSCC.

Alternative splicing (AS) is a critical factor for genome complexity and proteomic diversity 4. The abnormal patterns of AS are frequently found in cancer and starting to be recognized as an important player in tumor initiation and progression 5-7. For instance, the level of DOCK5 variant was notably increased in HNSCC. Overexpression of DOCK5 variant promoted the malignant behaviors of cancer cells, and *vice versa*. In addition, DOCK5 variant upregulation was closely associated with the unfavorable prognosis of HNSCC 8. The CD44 variant 4 and variant 6 were exclusively abundant in HNSCC, while variant 1 and variant 2 were found in normal oral keratinocytes. In addition, higher levels of variant 4 and variant 6 were especially detected in HNSCC cases with advanced stage or the cells with aggressive potential, suggesting that CD44 variant 4 and variant 6 might be important for HNSCC progression and metastasis 9.

The Cancer Genome Atlas (TCGA) and TCGASpliceSeq database are important bioinformatics platforms which provide valuable resources for data analysis. Previously we have constructed robust molecular signatures for predicting the prognosis of oral squamous cell carcinoma or oral precancerous lesions with mRNAs or microRNAs based on the TCGA database 10, 11. However, whether the alterations in the AS profile could be used to predict the clinical outcome of HNSCC remained unclear. In this study, we profiled the AS pattern in TCGA HNSCC cohort. Then a AS-based prognostic signature was developed based on the discovery cohort, and further validated with the validation cohort. More importantly, this prognostic signature-based nomogram model predicted the OS of HNSCC with extremely high accuracy, indicating it is a powerful prognostic tool for precision oncology and personalized medicine.

## Materials and Methods

### Public data source

The RNA-seq transcriptome data and the clinical information of TGCA HNSCC dataset were obtained from The National Cancer Institute Genomic Data Commons (NCI-GDC) (https://gdc.cancer.gov/). The alternative splicing data of TCGA HNSCC cohort was downloaded from TCGASpliceSeq database (https://bioinformatics.mdanderson.org /TCGASpliceSeq/). The Percent Spliced In (PSI) value, ranging from 0 to 100 (%), was used for quantifying AS events.

### AS profile identification and survival analysis

Upset plot was drawn to demonstrate the seven patterns of AS events (alternate acceptor site (AA), alternate donor site (AD), alternate promoter (AP), alternate terminator (AT), exon skip (ES), mutually exclusive exons (ME), and retained intron (RI)) in TCGA HNSCC cohort with UpsetR package in R. For each type of AS events, univariate Cox proportional hazard regression analysis was used to identify the OS-associated AS events. A prognostic signature was constructed for each type of AS events using the top 20 most significant OS-associated AS events in each corresponding pattern of AS events. Then the least absolute shrinkage and selection operator (LASSO) Cox regression model was used to select the optimal AS events into the multivariate Cox proportional hazards regression analysis. The multivariate analysis was performed to build up a risk score model. The ideal co-coefficients for the prognostic signatures were also determined. The beta value is the coefficient estimated by Cox analysis, which is equal to log (HR). A risk score for each patient was calculated as the sum of each AS's score, which was obtained by multiplying the PSI value of a AS event and its corresponding coefficient. Then the TCGA cohort was divided into high and low-risk groups base on the median value of the risk scores. The difference in OS between high and low-risk groups was evaluated by the Kaplan-Meier method and log-rank test.

### Prognostic signature construction and validation

The TCGA HNSCC cohort was randomly divided into the discovery cohort (n=244) and validation cohort (n=242) based on a computer-generated allocation sequence. The top 20 most significant OS associated AS events in seven patterns of AS events were subjected to LASSO Cox regression model analysis using the data of discovery cohort. Then a prognostic signature including five AS events was built up. The discovery cohort was stratified into high and low-risk group based on the median value of the risk scores. The OS between high and low-risk group in the discovery cohort was compared. Similarly, using the same scoring method, the risk scores were calculated for each patient in the validation cohort. The validation cohort was divided into high and low-risk groups using the same cut-off value as the discovery cohort. Then the difference in OS between high and low-risk group in the validation cohort was determined. Followed by combining risk score and other clinicopathological features into a multivariate model, univariate and multivariate analyses were performed to identify the independent predictors for HNSCC.

### Nomogram model construction

The risk score and other important clinicopathological parameters including age, gender, tumor grade and TNM stage were included to construct a nomogram model. Calibration plots were used to observe the prediction accuracy of the nomogram model.

### Splicing factor (SF)-AS regulatory network

The expression profiles of 404 SFs were obtained from the TCGA HNSCC dataset. Pearson correlation analysis was conducted to evaluate the association between the SFs and the prognosis-related AS events. Only factors satisfying the following conditions: *p* <0.05 and Pearson correlation coefficient >0.7 were chosen to build the SF-AS regulatory network with Cytoscape version 3.6.1.

### Statistical analysis

The independent t test or chi-square test were used to compare the differences of continuous or categorical variables between the discovery and validation cohorts. A *p* value of less than 0.05 was regarded as statistically significant.

## Results

### AS profiles in HNSCC

**Figure 1:**
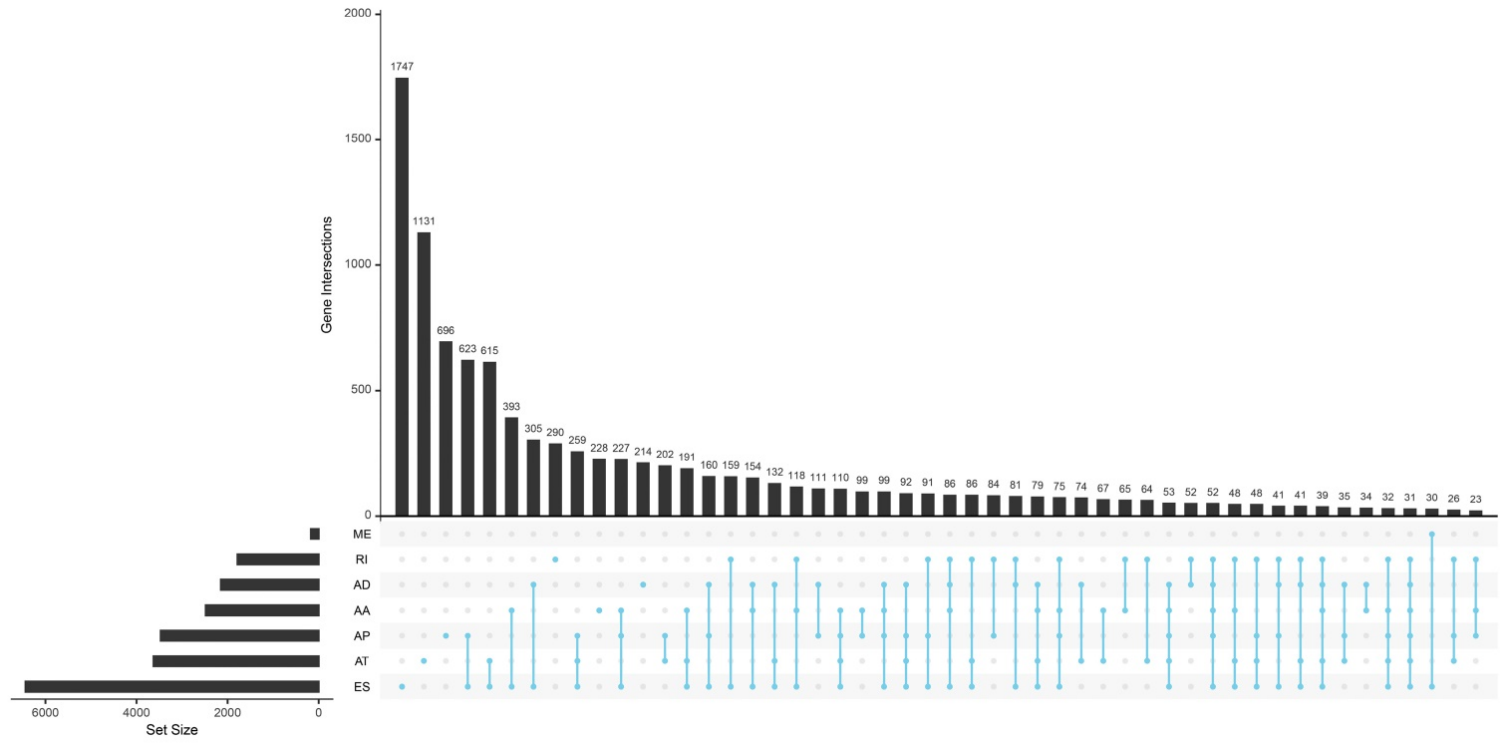
The upset plot of gene interactions among the seven types of AS events in TCGA HNSCC cohort.

Totally 42849 AS events of 10148 genes were found in TCGA HNSCC cohort. We detected 3500 AAs in 2482 genes, 3049 ADs in 2142 genes, 8598 APs in 3446 genes, 8309 ATs in 3609 genes, 16572 ESs in 6473 genes, 174 MEs in 174 genes and 2647 RIs in 1780 genes. It is common to observe that a single gene might have several AS patterns. However, no gene possesses the seven types of AS events simultaneously. The detailed information for the gene intersections among the seven types of AS events was showed in Figure 1.

### Identification of the OS-associated AS events

**Figure 2:**
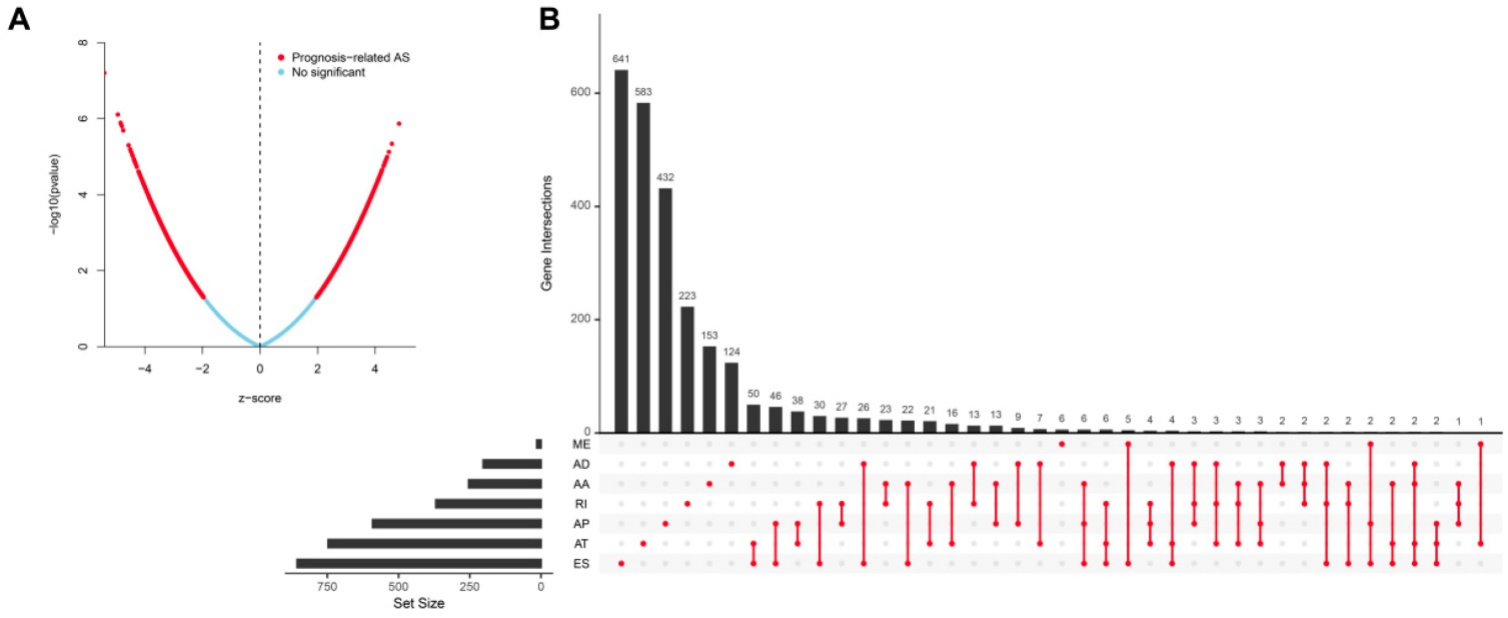
Identification of the OS-associated AS events. (A) The distribution of OS-associated AS events revealed by volcano plot. (B) The upset plot of gene interactions among the seven types of OS-related AS events.

**Figure 3:**
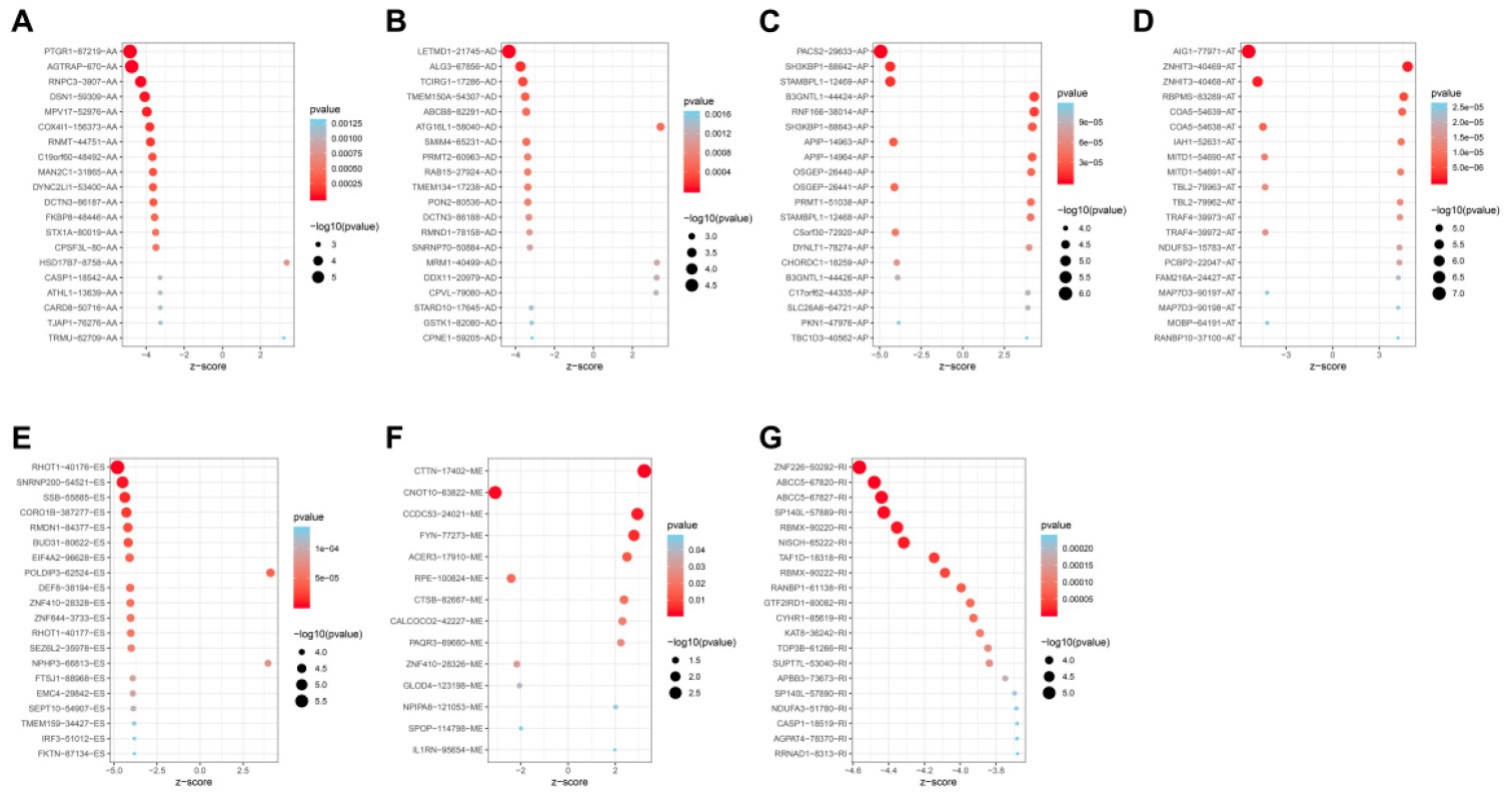
The most significant OS-associated AAs, ADs, APs, ATs, ESs, MEs and RIs in TCGA HNSCC cohort (A-G).

Univariate analysis identified a total of 4068-OS associated AS events in 2573 genes in TCGA HNSCC cohort. Among the OS-associated AS events, we found 265 OS-associated AAs in 253 genes, 218 OS-associated ADs in 203 genes, 908 OS-associated APs in 590 genes, 1166 OS-associated ATs in 746 genes, 1036-OS associated ESs in 859 genes, 14 OS-associated MEs in 14 genes and 461 OS-associated RIs in 368 genes. The volcano plot was used to observe the distribution of the OS-associated AS events (Figure 2A). Upset plot was drawn to visualize the gene intersections among the OS-associated AS events in Figure 2B. The most 20 most significant OS-associated AAs, ADs, APs, ATs, ESs and RIs were shown in Figure 3A-3E. As only 14 OS-associated MEs were identified, they were all depicted in Figure 3F.

### Construction of prognostic signatures based on each type of AS events

**Figure 4:**
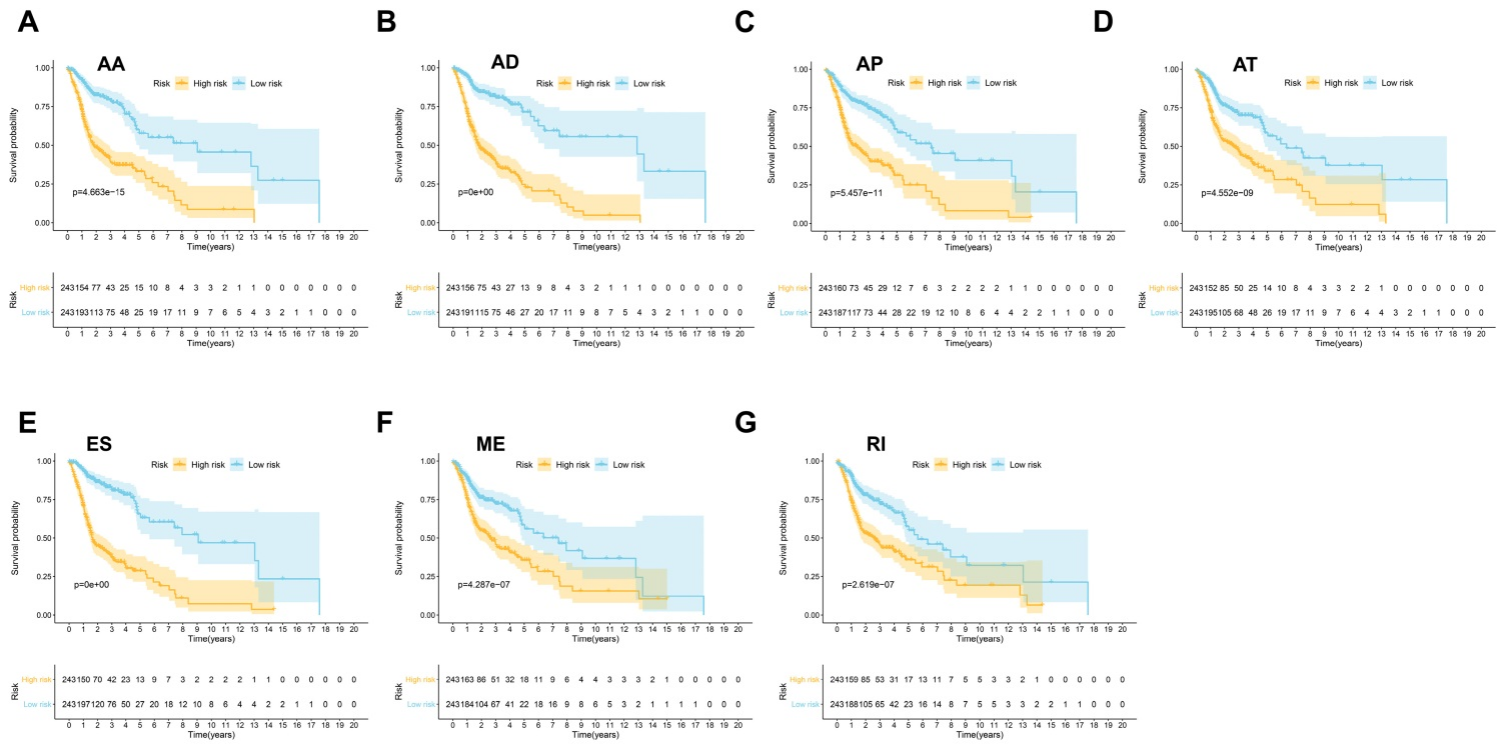
The prognostic signatures built on each type of AS events (AA, AD, AP, AT, ES, ME and RI) (A-G).

Then seven prognostic signatures were built up based on each type of AS events. The detailed risk score formulas were summarized in Supplementary Table 1. The HNSCC patients were stratified into high and low-risk groups based on the AA prognostic signature, our results demonstrated that the HNSCC patients in the high-risk group suffered significantly shorter OS than those in the low-risk group (*p*=4.663e-15) (Figure 4A). Similar findings were observed with prognostic signatures based on AD (*p*=0e+00), AP (*p*=5.457e-11), AT (*p*=4.552e-09), ES (*p*=0e+00), ME (*p*=4.287e-17) or RI (*p*=2.619e-07) (Figure 4B-4G).

### Development and validation of a AS-based prognostic signature

**Figure 5:**
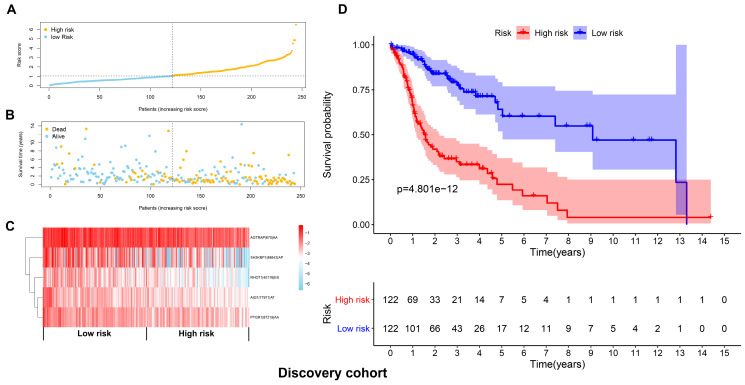
Development of a prognostic signature based on the seven types of AS events in the discovery cohort. (A) The distributions of risk scores in the two groups. (B) The distributions of OS and OS status between two groups. (C) The PSI values of the five prognostic AS events in the high and low-risk groups. (D) The HNSCC cases in the high-risk group had significant shorter OS than those in the low-risk group.

**Figure 6:**
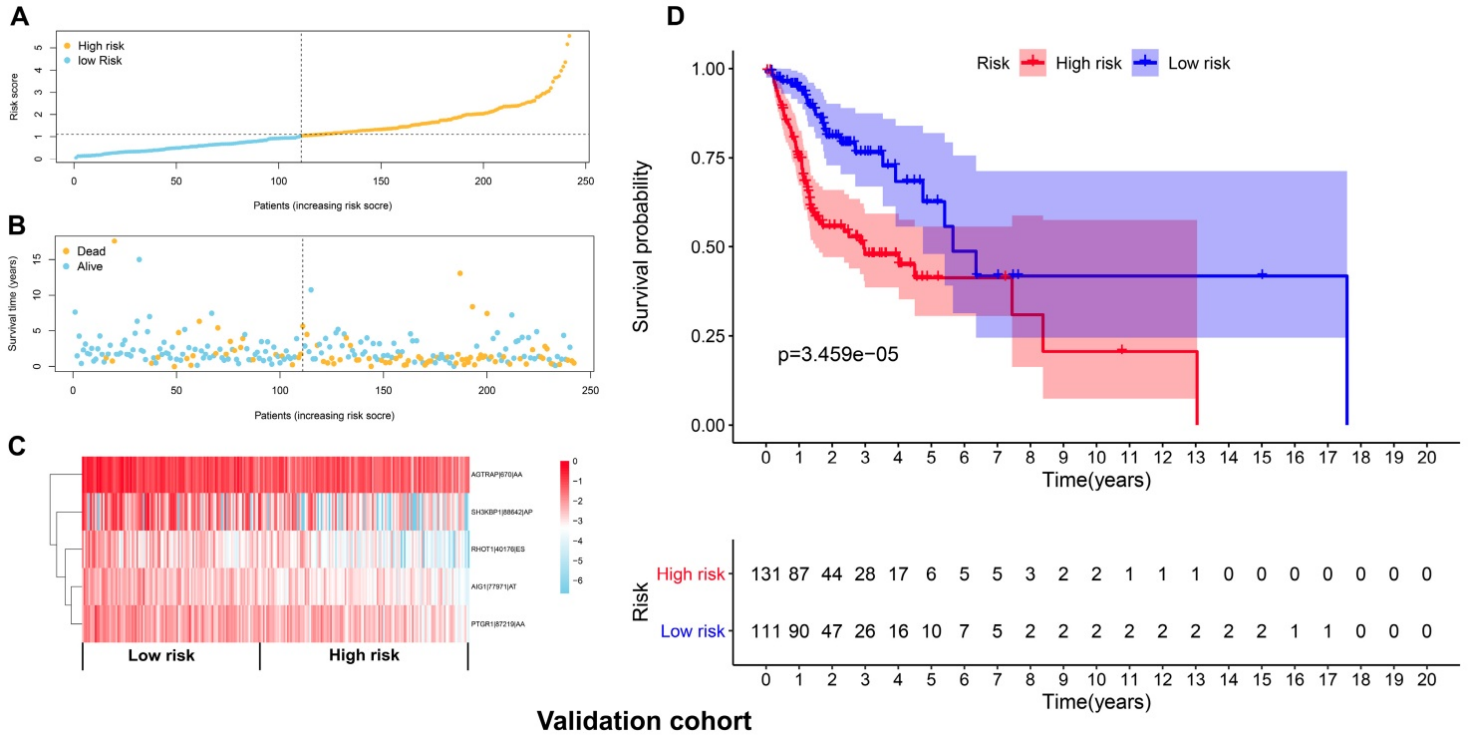
Validation of the prognostic signature with the validation cohort. (A) The distributions of risk scores in the two groups. (B) The distributions of OS and OS status between two groups. (C) The PSI values of the five prognostic AS events in the high and low-risk groups. (D) The OS was notably shorter in high-risk group than in the low-risk group.

The top 20 OS-associated AS events were subjected to LASSO Cox regression model analysis and multivariate analysis to build up a prognostic signature (AIG1|77971|AT, PTGR1|87219|AA, RHOT1|40176|ES, AGTRAP|670|AA, SH3KBP1|88642|AP) using the discovery cohort. The formula for calculating the risk scores was as follows: risk scores=(AIG1|77971|AT×-4.09) + (PTGR1|87219|AA×-3.31) + (RHOT1|40176|ES×-3.89) + (AGTRAP|670|AA×-2.45 + SH3KBP1|88642|AP×-1.45). The discovery cohort was divided into high and low-risk groups based on the median value of the risk scores. Figure 5A and 5B demonstrated the distributions of risk scores, and the distributions of OS and OS status, respectively. The pattern of five prognostic AS events between high and low- risk groups was shown in Figure 5C. The survival analysis revealed that the HNSCC patients in the high-risk group had remarkably shorter OS than those in the low-risk group (*p*=4.801e-12) (Figure 5D). Similarly, Figure 6A and 6B demonstrated the distributions of risk scores, and the distributions of OS and OS status in the validation cohort, respectively. The PSI values of five prognostic AS events between high and low risk groups were revealed by heatmap (Figure 6C). For the validation cohort, the OS was also significantly shorter in the high-risk group compared to the low-risk group (*p*=3.459e-05).

### The AS-based prognostic signature was an independent prognostic indicator for HSNCC

**Figure 7:**
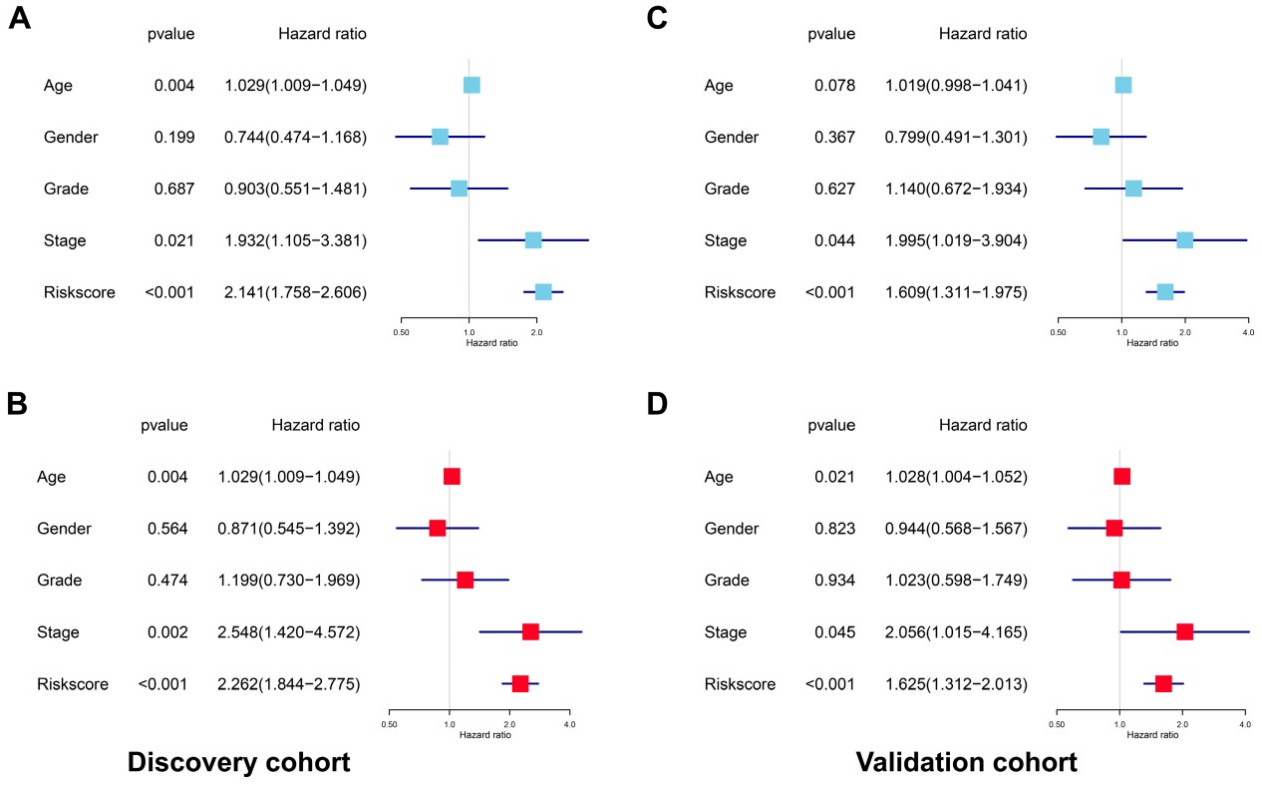
The prognostic signature was an independent indicator in HNSCC. (A) Univariate analysis revealed the clinicopathological factors strongly correlated with OS in the discovery cohort. (B) Multivariate analysis showed that the age, stage and risk score were the independent indicators for HNSCC in the discovery cohort. (C) Univariate analysis showed the clinicopathological factors notably correlated with OS in the validation cohort. (D) Multivariate analysis demonstrated that the age, stage and risk score were the independent indicators for HNSCC in the validation cohort.

After deleting cases with missing values in age, gender, tumor grade or TNM stage, a total of 201 and 204 cases remained in the discovery cohort and validation cohort, respectively. No significant differences were found for the clinicopathological parameters between discovery cohort and validation cohort (Supplementary Table 2 and Supplementary Table 3). For the discovery cohort, the univariate analysis showed that age (*p*=0.004, HR=1.029, 95% CI=1.009-1.049), stage (*p*=0.021, HR=1.932, 95% CI=1.105-3.381) and risk score (*p*<0.001, HR=2.141, 95% CI=1.758-2.606) were significantly associated with OS (Figure 7A). The multivariate analysis revealed that age (*p*=0.004, HR=1.029, 95% CI=1.009-1.049), stage (*p*=0.002, HR=2.548, 95%CI=1.420-4.572) and risk score (*p*<0.001, HR=2.262, 95% CI=1.844-2.775) were independent prognostic indicators for HNSCC (Figure 7B). For the validation cohort, the univariate analysis showed that stage (*p*=0.044, HR=1.995, 95% CI=1.019-3.904) and risk score (*p*<0.001, HR=1.609, 95% CI=1.311-1.975) were significantly associated with OS (Figure 7C). The multivariate analysis revealed that age (*p*=0.021, HR=1.028, 95% CI=1.004-1.052), stage (*p*=0.045, HR=2.056, 95% CI=1.015-4.165) and risk score (*p*<0.001, HR=1.625, 95% CI=1.312-2.013) were independent prognostic indicators for HNSCC (Figure 7D).

### Nomogram model construction and prediction

**Figure 8:**
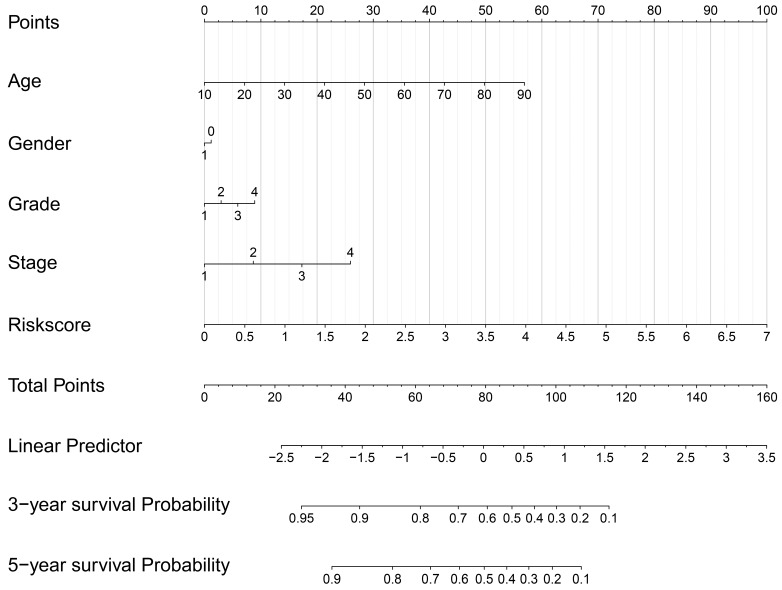
Construction of a prognostic signature-based nomogram model.

**Figure 9:**
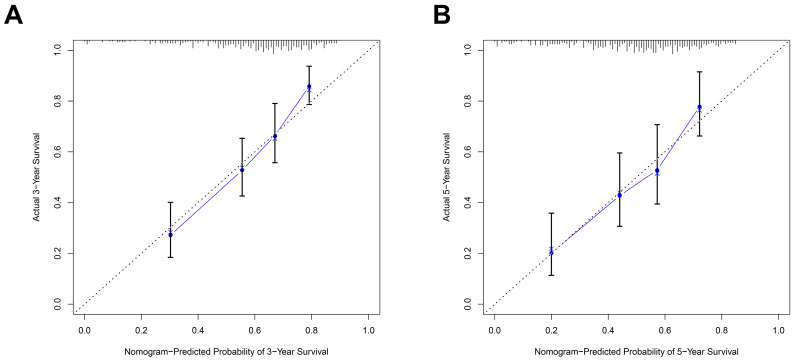
The predictive power of the prognostic signature-based nomogram mode evaluated by calibration curves. The calibration plots demonstrated that the nomogram model exhibited excellent performance for predicting the 3-year OS (A) and 5-year OS (B).

As the above AS-based prognostic signature showed great promise for predicting the prognosis of HNSCC, a more convenient and sensitive nomogram model which included the AS-based prognostic signature and other clinicopathological factors was developed. As shown in Figure 8, the 3-year OS or 5-year OS of each HNSCC patient could be easily predicted by calculating the total nomogram score. The calibration plots were drawn to evaluate the reliability of the nomogram model. Oure results showed that the nomogram model predicted outcome was almost overlapping with the actual outcome for both 3-year OS or 5-year OS of HNSCC (Figure 9A-9B).

### The SF-AS interaction network

**Figure 10:**
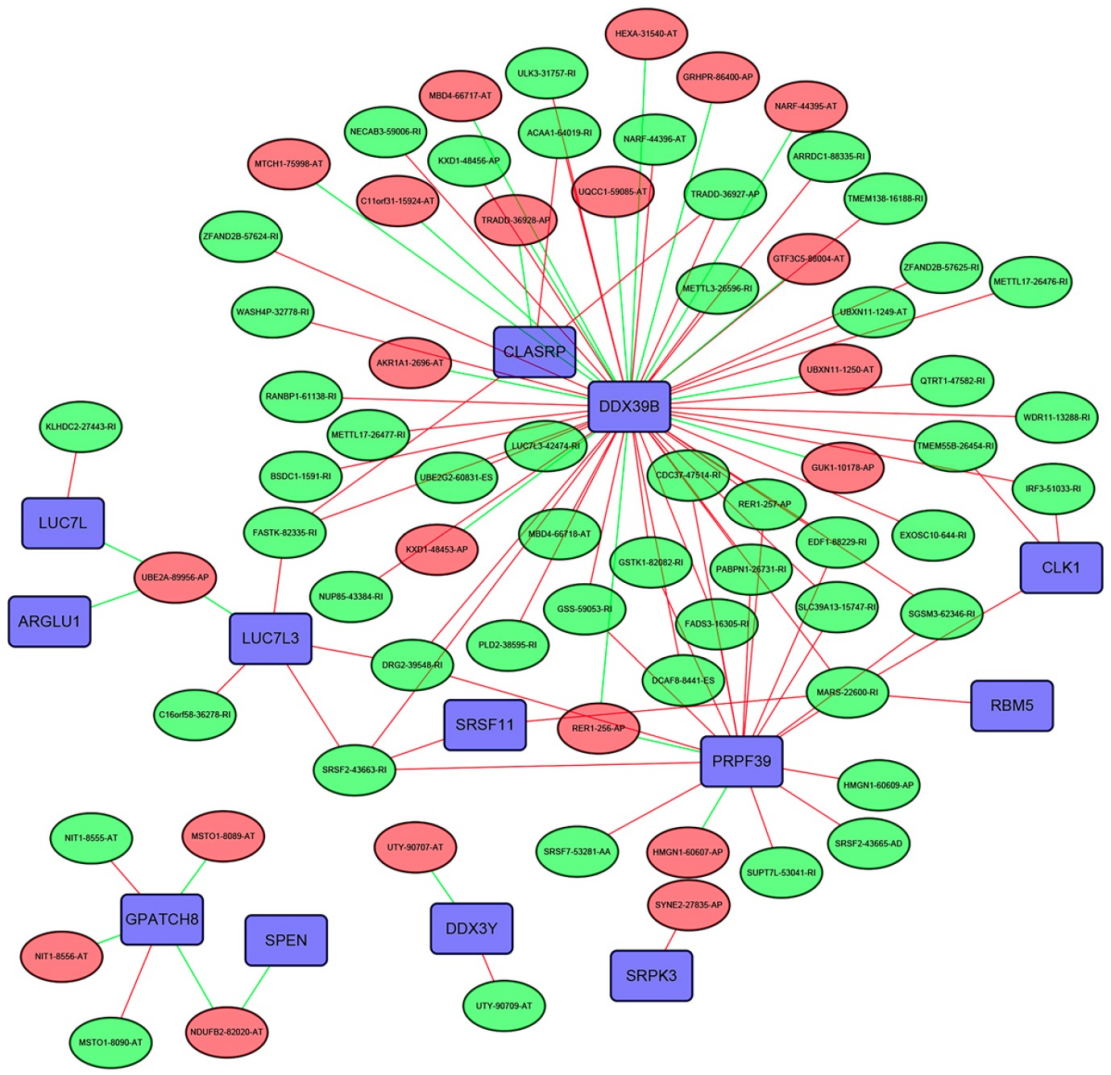
The interaction network between SFs and OS-associated AS events. The red or green ellipses represented the AS events positively or negatively correlated with OS. Violet rectangles indicated SFs. The positive/negative correlations (r>0.7 or r<-0.7) between SFs and AS events were indicated with red/green lines.

The Pearson correlation analysis was performed to identified the most correlated OS-associated AS events and SFs. Only the SF-AS interactions with the coefficients larger than 0.7 were included in the Figure 10. Our results showed that many SFs such as DDX39B, CLASRP and PRPF39 might play important roles in regulating the changes of AS events.

## Discussion

Development of molecular markers for accurately predicting the clinical outcome of HNSCC is of great significance. An effective and reliable prognostic signature provides personalized, risk-directed treatment selection in everyday clinical management 12. In this study, we have profiled a number of OS-associated AS events in the TCGA HNSCC cohort. In addition, the prognostic signature built on each type of AS events divided the TCGA HNSCC cohort into high and low risk groups with obviously different OS. Moreover, a risk signature including five AS events was built on seven types of AS events with the discovery cohort, and closely associated with OS of HNSCC. More importantly, this AS-based prognostic signature was robustly validated in the validation cohort. To make this risk signature more convenient for potential clinical application, a nomogram model based on this prognostic signature was developed, and high agreement was found between nomogram model predicted outcome and actual clinical outcome. Furthermore, the most correlated SF-AS interactions in the TCGA HNSCC cohort were identified.

This might be the first study to construct and validate a robust AS-based prognostic signature for HNSCC. Although Xing et al revealed some prognosis-related AS events and built up some prognostic models in HNSCC, they did not validate their findings 13. In addition, the reliability and effectiveness of their prediction models was unknown. Similar with our findings, altered AS profiles has been found to be associated with prognosis in many types of cancers. For instance, a total of 3691 and 2403 AS events were found to be significantly correlated with survival in lung adenocarcinoma and lung squamous cell carcinoma 14. Similarly, a robust prognostic prediction model which included seven AS events showed great promise for predicting OS of patients with kidney renal clear cell carcinoma 15.

Normal alternative splicing plays an essential role in regulating many biological processes such as proliferation, growth, differentiation and development 16. Abnormal splicing can generate protein isoforms that promote the formation and progression of tumor as well as resistance to therapy 17, 18. SFs are the important regulators for the alternative splicing. Therefore, it is common to observe that deregulation of SFs is closely involved in tumor development 19, 20. Our results showed that DDX39B seemed to be a central node in the SF-AS interaction network. Awasthi et al revealed that DDX39B was important for maintaining the normal levels of pre-ribosomal RNA by controlling its stability and synthesis. In addition, DDX39B was frequently upregulated in many cancer types and promoted the proliferative capacity of cells 21. Activation of androgen receptor (AR) splice variants, especially AR-V7, are associated with unfavorable clinical outcome of prostate cancer. DDX39B was demonstrated to be an upstream regulator of AR-V7, indicating that it plays a critical role in regulating the progression of prostate cancer 22.

One possible limitation of our study is that the AS-based prognostic signature needs further validation by external independent studies. In addition, deeper exploration is warranted to understand the biological functions of the prognosis related-AS events in the initiation and progression of HNSCC. Moreover, the major racial categories of TCGA HNSCC cohort are white and black people. Whether our AS-based prognostic signature also accurately predicts the prognosis of HNSCC in other races needs further investigation. Furthermore, it is still difficult to apply the AS-based prognostic signature in the clinical setting. Firstly, detecting the expression of AS events for most HNSCC patients is impossible due to the costly high throughput RNA-seq approach. Secondly, the RNA-seq is not commonly used for clinical detection. The rapid development of sequencing technologies and methods will hopefully make it possible to use the AS-based prognostic signatures for clinical applications.

Collectively, we have identified the OS-associated AS events in HNSCC. In addition, a robust AS-based prognostic signature is successfully built up to accurately predict the OS of HNSCC, which might contribute to facilitate personalized clinical management of patients with HNSCC.

## Supplementary Material

Supplementary tables.

## Abbreviations

AS: alternative splicing
HNSCC: head and neck squamous cell carcinoma
OS: overall survival
AA: alternate acceptor site
AD: alternate donor site
AP: alternate promoter
AT: alternate terminator
ES: exon skip
ME: mutually exclusive exons
RI: retained intron
TCGA: The Cancer Genome Atlas
PSI: percent spliced in
SF: splicing factor
LASSO: least absolute shrinkage and selection operator
TNM: tumor, node, metastasis
