# Supplementary material for: Identification and validation of an alternative splicing-based prognostic signature for head and neck squamous cell carcinoma
Source: J Cancer. 2020 May 18;11(15):4571–80. doi: 10.7150/jca.44746 (PMC7255372; doi:10.7150/jca.44746)
Supplement: Supplementary file 1 — Supplementary tables. [file jcav11p4571s1.pdf]

Supplementary Table 1. The prognostic signatures based on each type of AS events.

AS type (AA)	coef	AS type (AD)	coef
PTGR1|87219|AA	-3.78	ALG3|67856|AD	-1.22
AGTRAP|670|AA	-2.15	TCIRG1|17286|AD	-6.14
DSN1|59309|AA	-18.44	TMEM150A|54307|AD	-2.18
MPV17|52976|AA	-1.01	ATG16L1|58040|AD	5.81
RNMT|44751|AA	-2.26	SMIM4|65231|AD	-5.31
C19orf60|48492|AA	-2.81	PON2|80536|AD	-2.32
FKBP8|48446|AA	-3.51	RMND1|78158|AD	-2.50
STX1A|80019|AA	-5.49	SNRNP70|50884|AD	-1.16
HSD17B7|8758|AA	1.85	MRM1|40499|AD	11.35
CASP1|18542|AA	-3.23	DDX11|20979|AD	2.60
TJAP1|76276|AA	-1.84	CPVL|79080|AD	3.76
		CPNE1|59205|AD	-1.55
**AS type (AP)**	**coef**	**AS type (AT)**	**coef**
PACS2|29633|AP	-4.78	AIG1|77971|AT	-5.20
SH3KBP1|88642|AP	-1.53	RBPMS|83289|AT	2.77
STAMBPL1|12469|AP	-1.59	FAM216A|24427|AT	4.11
APIP|14963|AP	-3.78	MOBP|64191|AT	-1.67
C5orf30|72920|AP	-3.45		
PKN1|47976|AP	-1.10		
**AS type (ES)**	**coef**	**AS type (ME)**	**coef**
RHOT1|40176|ES	-2.86	CNOT10|63822|ME	-2.59
CORO1B|387277|ES	-0.78	FYN|77273|ME	1.25
RMDN1|84377|ES	-4.83	CTSB|82667|ME	8.29
POLDIP3|62524|ES	21.94	CALCOCO2|42227|ME	14.90
DEF8|38194|ES	-1.81	PAQR3|69660|ME	3.29
ZNF644|3733|ES	-2.12	ZNF410|28326|ME	-2.25
SEZ6L2|35978|ES	-4.00	GLOD4|123198|ME	-1.36
FTSJ1|88968|ES	-7.51		
EMC4|29842|ES	-4.88		
SEPT10|54907|ES	-1.86		
TMEM159|34427|ES	-1.05		
FKTN|87134|ES	-2.19		
**AS type (RI)**	**coef**		
ZNF226|50292|RI	-2.72		
ABCC5|67820|RI	-0.97		
RBMX|90220|RI	-1.29		
KAT8|36242|RI	-3.80		
CASP1|18519|RI	-1.02		

Supplementary Table 2. **The clinical information of the discovery cohort**.

Clinicopathological features	Number
**Age**	
Mean (SD)	61.11 (12.07)
**Gender, n (%)**	
Male	145 (72.14%)
Female	56 (27.86%)
**Pathological diagnosis**	
Squamous cell carcinoma	201 (100%)
**Tumor grade**	
G1	27 (13.43%)
G2	127 (63.18%)
G3	47 (23.38%)
G4	0 (0.00%)
**TNM stage**	
Stage I	12 (5.97%)
Stage II	33 (16.42%)
Stage III	38 (18.91%)
Stage IV	118 (58.71%)

Supplementary Table 3. **The clinical information of the validation cohort**.

Clinicopathological features	Number
**Age**	
Mean (SD)	61.57 (12.03)
**Gender, n (%)**	
Male	146 (71.57%)
Female	58 (28.43%)
**Pathological diagnosis**	
Squamous cell carcinoma	204 (100%)
**Tumor grade**	
G1	28 (13.73%)
G2	126 (61.76%)
G3	49 (24.02%)
G4	1 (0.05%)
**TNM stage**	
Stage I	13 (6.37%)
Stage II	32 (15.69%)
Stage III	35 (17.16%)
Stage IV	124 (60.78%)
